# Image-Based Molecular Phenotyping of Pancreatic Ductal Adenocarcinoma

**DOI:** 10.3390/jcm9030724

**Published:** 2020-03-07

**Authors:** Georgios A. Kaissis, Sebastian Ziegelmayer, Fabian K. Lohöfer, Felix N. Harder, Friederike Jungmann, Daniel Sasse, Alexander Muckenhuber, Hsi-Yu Yen, Katja Steiger, Jens Siveke, Helmut Friess, Roland Schmid, Wilko Weichert, Marcus R. Makowski, Rickmer F. Braren

**Affiliations:** 1 Technical University of Munich, School of Medicine, Department of Diagnostic and Interventional Radiology, 81675 Munich, Germany; g.kaissis@tum.de (G.A.K.); ga89rog@mytum.de (S.Z.); fabian.lohoefer@tum.de (F.K.L.); felix.harder@tum.de (F.N.H.); friederike.jungmann@tum.de (F.J.); daniel.sasse@tum.de (D.S.); marcus.makowski@tum.de (M.R.M.); 2 Imperial College of Science, Technology and Medicine, Faculty of Engineering, Department of Computing, SW7 2AZ London, UK; 3 Technical University of Munich, School of Medicine, Institute for Pathology, 81675 Munich, Germany; Alexander.Muckenhuber@tum.de (A.M.); hsi-yu.yen@tum.de (H.-Y.Y.); katja.steiger@tum.de (K.S.); wilko.weichert@tum.de (W.W.); 4 Institute of Developmental Cancer Therapeutics, West German Cancer Center, University Hospital Essen, 45147 Essen, Germany; Jens.Siveke@uk-essen.de; 5 Division of Solid Tumor Translational Oncology, German Cancer Consortium (DKTK, parter site Essen, Germany) and German Cancer Research Center, DKFZ, 69120 Heidelberg, Germany; 6 Technical University of Munich, School of Medicine, Surgical Clinic and Policlinic, 81675 Munich, Germany; helmut.friess@tum.de; 7 Technical University of Munich, School of Medicine, Department of Internal Medicine II, 81675 Munich, Germany; direktion.med2@mri.tum.de

**Keywords:** radiomics, pancreatic cancer, molecular subtypes

## Abstract

To bridge the translational gap between recent discoveries of distinct molecular phenotypes of pancreatic cancer and tangible improvements in patient outcome, there is an urgent need to develop strategies and tools informing and improving the clinical decision process. Radiomics and machine learning approaches can offer non-invasive whole tumor analytics for clinical imaging data-based classification. The retrospective study assessed baseline computed tomography (CT) from 207 patients with proven pancreatic ductal adenocarcinoma (PDAC). Following expert level manual annotation, Pyradiomics was used for the extraction of 1474 radiomic features. The molecular tumor subtype was defined by immunohistochemical staining for KRT81 and HNF1a as quasi-mesenchymal (QM) vs. non-quasi-mesenchymal (non-QM). A Random Forest machine learning algorithm was developed to predict the molecular subtype from the radiomic features. The algorithm was then applied to an independent cohort of histopathologically unclassifiable tumors with distinct clinical outcomes. The classification algorithm achieved a sensitivity, specificity and ROC-AUC (area under the receiver operating characteristic curve) of 0.84 ± 0.05, 0.92 ± 0.01 and 0.93 ± 0.01, respectively. The median overall survival for predicted QM and non-QM tumors was 16.1 and 20.9 months, respectively, log-rank-test *p* = 0.02, harzard ratio (HR) 1.59. The application of the algorithm to histopathologically unclassifiable tumors revealed two groups with significantly different survival (8.9 and 39.8 months, log-rank-test *p* < 0.001, HR 4.33). The machine learning-based analysis of preoperative (CT) imaging allows the prediction of molecular PDAC subtypes highly relevant for patient survival, allowing advanced pre-operative patient stratification for precision medicine applications.

## 1. Introduction

In pancreatic ductal adenocarcinoma (PDAC), several lines of evidence suggest the existence of distinct subtypes with prognostic and predictive relevance. For example, Breast Cancer Gene (BRCA) 1/2 mutations have been identified in a subgroup of PDAC, and these patients exhibit an improved therapy response to the poly (ADP-ribose) polymerase (PARP) inhibitor Olaparib [1]. The majority of PDAC can be classified into two distinct subtypes based on transcriptome profiling and immunohistochemical staining of cytokeratin-81 (KRT81) and hepatocyte nuclear factor-1A (HNF1a) [2,3,4]: a so-called non-quasi-mesenchymal (non-QM), i.e., classical, epithelial KRT81-/HNF1a-subtype exhibiting slightly improved survival and therapy response, notably to the FOLFIRINOX regimen, while not responding as well to gemcitabine-based treatment. The so-called quasi-mesenchymal (QM), basal-like or KRT81+/HNF1a- subtype has a dismal overall survival and resistance towards virtually all currently employed therapy regimens. However, the QM subtype does exhibit a superior response to gemcitabine in comparison to the non-QM subtype [5]. These findings support differential treatment of patients based on individual molecular tumor make-up. Hitherto this has proven infeasible in clinical routine because of individual tumor heterogeneity and the immanent sampling errors of biopsy and lack of clinically suitable and sufficiently robust transcriptomic assays.

Recent developments in machine learning (ML)-based medical image analysis such as Radiomics provide encouraging examples of molecular phenotyping from imaging data. For instance, the non-invasive genetic profiling of lung carcinoma has been recently demonstrated [6], and imaging biomarkers have recently been shown to outperform the risk metrics defined in the current WHO classification of gliomas [7].

We recently reported on machine-learning approaches for the prediction of molecular subtypes and survival risk in PDAC patients from pre-operative magnetic resonance imaging (MRI) [8,9]. We noted that limited availability of MR imaging data, overall reduced image quality and the less-quantitative and unstandardized nature of MRI pose barriers to algorithm development and generalization.

To enhance clinical translation, we extend our previous results to computed tomography (CT) by training and validating an algorithm capable of discriminating between the QM and the non-QM subtypes of PDAC with high performance based on pre-operative CT imaging in a therapy-naïve surgical cohort of PDAC patients. We next applied this algorithm to histopathologically so-called unclassifiable, KRT81+/HNF1a+ tumors resulting in significant separation of overall survival time, suggesting identification of clinically distinct subgroups not identified by traditional histology.

## 2. Experimental Section

The study was designed as a retrospective cohort study. The STROBE checklist [10] and patient recruitment flowchart are included in the Supplementary Materials. Institutional review board approval was obtained for the study, waiving the requirement for individual informed consent. All analyses were carried out in accordance with pertinent laws and regulations and in conformity with the Helsinki Declaration. The study was approved by the Ethics Committee of the Technical University of Munich, School of Medicine (Protocol Number 180/17S; date of approval: 9 May 2017).

Participants were screened for eligibility based on a search of the hospital picture archiving system (PACS) for portal-venous-phase CT scans (70 s post injection of iodinated contrast media) including the pancreatic region from October 2006 to March 2019. A total of 237 candidates were confirmed eligible based on histologically ascertained pancreatic adenocarcinoma. Of these, 30 were excluded from the analysis due to insufficient technical quality of the CT scan (including motion artifacts and significant beam hardening due to nearby foreign materials), pre-existing malignant disease or any previous therapy, including chemotherapy, or loss to follow-up earlier than 2 weeks post-operatively. The 207 resulting patients were separated into two subcohorts: cohort A, including the patients whose immunohistochemical assessment resulted in an unequivocal classification as QM or non-QM (*n* = 181), and cohort B, including the patients with histopathologically unclassifiable double positive (KRT81+/HNF1a+) tumors (*n* = 26), as described below.

The patients underwent computed tomography on the following CT scanner models: Siemens Somatom Definition (*n* = 87, 64-row, Siemens Healthineers, Erlangen, Germany), Philips iCT (*n* = 79, 256-row, Philips Healthcare, Best, The Netherlands), Philips IQON Spectral CT (*n* = 41, 64-row, Philips Healthcare, Best, The Netherlands). Clinical data collection and follow-up were handled by the departments of surgery and gastrointestinal oncology at our institution and ended on the 31st of March, 2019. The clinical variables collected were: Age, Sex, pTNM according to UICC 6th edition (pT: tumor stage, pN: nodal status, M: metastasis), R (resection margin), G (tumor grading), first-line adjuvant chemotherapy regimen, baseline CA19-9 (carbohydrate antigen 19-9), baseline CEA (carcinoembryonic antigen), tumor location (head/ body vs. tail) and overall survival.

Tumors were segmented independently under standardized conditions by two experts with 3- and 5-year experience in abdominal radiology, quality-controlled or corrected by a third expert with 8 years of experience in abdominal radiology and pancreatic imaging. After a period of two weeks, 20 randomly selected datasets from the three groups were sampled, randomly shuffled and presented to the same observers for re-segmentation. Segmentation was performed using the segmentation tool ITK-SNAP [11]. Radiomic features were extracted using PyRadiomics [12] using the settings detailed in the Supplementary Materials and normalized to the (0,1) interval. In total, 1474 radiomic features were extracted. Of these, features with missing values, all-null values, zero variance, features unstable to between-observer segmentation or to segmentation and re-segmentation (based on an intra-class correlation coefficient [13] below 0.9) were eliminated from the analysis. The remaining 161 features were normalized by tumor volume (calculated by PyRadiomics as mesh volume) as suggested by [14]. A Random Forest machine-learning algorithm [15] was used to model the features using the settings detailed in the Supplementary Materials with target labels of QM (KRT81+/HNF1a-) or non-QM (KRT81-/HNF1a- or KRT81-/HNF1a+). To alleviate class imbalance, per-sample weighting inversely proportional to the class population was applied. The classification performance with respect to the labels was assessed using sensitivity, specificity and ROC-AUC (area under the receiver operating characteristic curve) metrics using five-fold shuffle-split cross-validation with a test sample fraction of 0.3. Feature importance was assessed by reduction in Gini impurity [16] for each of the five folds and the average feature importance and standard deviation are reported. The algorithm achieving the highest ROC-AUC in cross-validation was applied to the cohort of unclassifiable PDAC, and the resulting predicted labels used as inputs for successive survival modelling. A technical evaluation of the study according to the recently published RSNA criteria [17] can be found in the Supplementary Materials.

To address bias associated with clinical covariates, cross-tabulations and multivariate Cox proportional hazards testing were performed. Univariate overall survival was modelled using the Kaplan Meier method including any censorship. The chi-squared-test was used for cross-tabulations, Students t-test for continuous variables and the log-rank-test for survival comparisons. A two-sided significance level of *p* < 0.05 was chosen.

**Figure 1:**
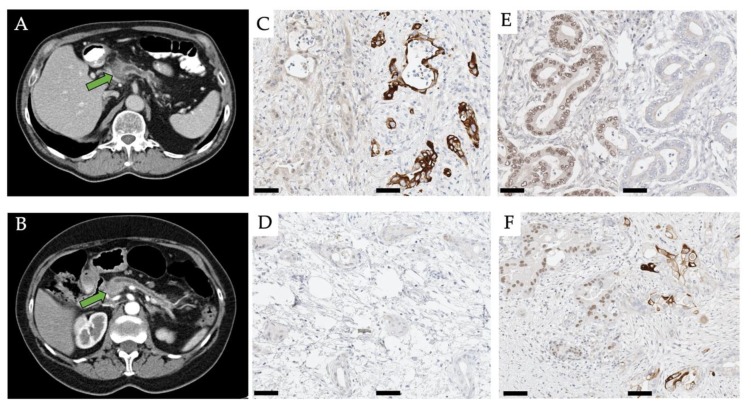
CT images of a patient with a QM (**A**) and a non-QM (**B**) PDAC in the pancreatic head (arrow). Window level 36 Hounsfield-Unit width 350 Hounsfield-Unit in both cases. Micro-photographs of representative immunohistochemical specimens of a HNF1a-/KRT81+ (QM) tumor (**C**), a HNF1a-/KRT81- (non-QM) tumor (**D**), a HNF1a+/KRT81- (non-QM) tumor (**E**) and a HNF1a+/KRT81+ (unclassifiable) tumor (**F**). Scale bar 50 µm. HNF/KRT immunostainings left/right in each subfigure, respectively.

Histopathological staining and immunohistochemical workup were performed by application of surrogate markers to determine the molecular subtype of PDAC based on the previously established immunohistochemical protocol described in [3]. Briefly, 2 µm sections were stained for KRT81 and HNF1a, and tumors were categorized into either one of three classes based on a cut-off value of 30% for tumor cell positivity/negativity: KRT81+/HNF1a- tumors were designated QM, KRT81-/HNF1a- and KRT81-/HNF1a+ tumors were grouped as non-QM. KRT81+/HNF1a+ tumors were designated double positive, i.e., unclassifiable. Classification was performed separately by two expert pathologists with 8 and 12 years of experience and quality-controlled or corrected by a third pathologist with 18 years of experience. Exemplary micro-photographs of the immunohistochemical stains alongside representative CT images can be found in Figure 1.

## 3. Results

**Figure 2:**
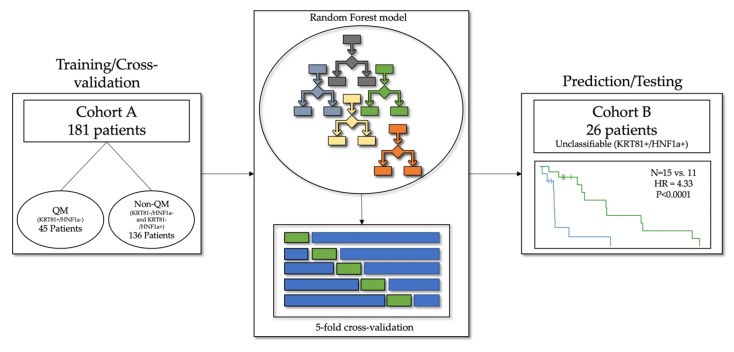
In total, 207 patients were included in the study. Among them, 181 patients in cohort A with confirmed QM and non-QM tumors served as the training and cross-validation data, and 45 patients in cohort B with unclassifiable tumors were used for model testing.

In total, 207 patients were included in the study, 181 in the training/cross-validation cohort A and 26 in the testing cohort B. A schematic representation of the cohorts can be found in Figure 2.

To assess relevant clinical covariates and confounders, clinical parameter evaluation and cross-tabulations were performed to assess the associated parameter distributions. These are found in Table 1.

No statistically significant difference was observed in the distribution of clinical confounding variables between the cohorts and none of the parameters were significantly associated with overall survival in multivariate survival modelling (Supplementary Figures S1 and S2).

**Figure 3:**
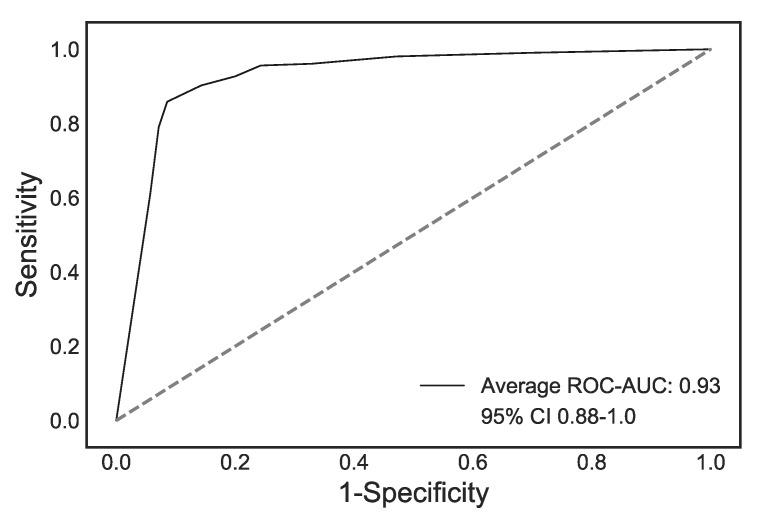
Average ROC curve (black) and 95% confidence interval (CI) (shaded blue) for the five cross-validation folds.

In total, 1474 features were extracted from the CTs, of which 161 remained after feature engineering. The Random Forest algorithm was trained using the five-fold cross-validation approach detailed above on cohort A, and achieved a sensitivity of 0.84 ± 0.05, a specificity of 0.92 ± 0.01 and a ROC-AUC of 0.93 ± 0.01 for the classification of QM vs. non-QM tumors across the five folds. The ROC curves for cohort A and the average ROC curve are shown in Figure 3.

Feature importance assessment was performed using the *Gini* impurity index, measuring the quality of the split in each node of the random forest trees. The importance averages were averaged across folds, and the 20 features with the highest importance are presented in Supplementary Table S1 sorted in descending order. Among these features, several represent the spectrum of image homogeneity/heterogeneity, notably Entropy-/Energy-, Uniformity/Non-Uniformity and Correlation-/Variance-related features.

**Figure 4:**
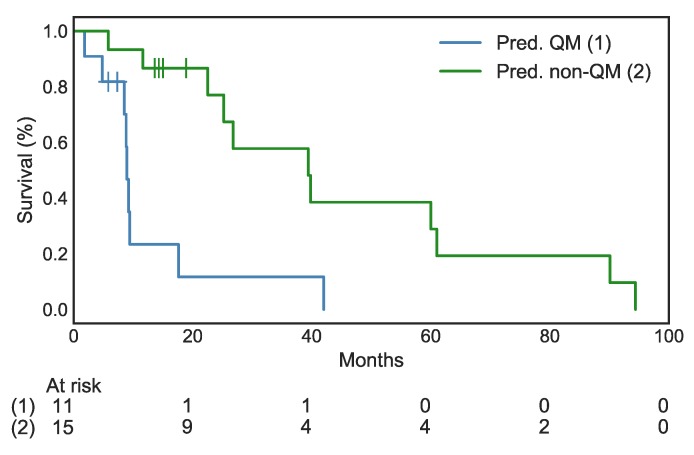
Highly significant separation of overall survival in the groups with predicted QM vs. non-QM tumors. HR: 4.33, 95% CI 1.14–13.32, log-rank test *p* < 0.0001. Vertical ticks indicate censorship.

The trained Random Forest algorithm was applied to the previously unseen data of cohort B, consisting of histopathologically unclassifiable “double positive” tumors. The algorithm predicted a label of QM or non-QM for 12 and 14 out of 26 patients, respectively. Kaplan-Meier survival analysis of these cases resulted in a highly significant separation of the two predicted cohorts, with a median survival of 8.9 months for the predicted QM cases and of 39.8 months for the predicted non-QM cases (log-rank-test *p* < 0.0001, HR 4.33, 95% CI 1.41–13.32, Figure 4).

## 4. Discussion

Here, we present a machine-learning algorithm capable of distinguishing between image-derived phenotypes representative of immunohistochemically defined molecular subtypes of PDAC. The application of this algorithm to histopathologically unclassifiable tumors identifies two patient groups with significantly different overall survival. We therefore hypothesize that the algorithm is capable of re-identifying the dominant features of the QM and non-QM molecular subtypes in CT images and that radiomics-based phenotyping may thus offer a clinically usable classification advantageous over histopathology in the notoriously heterogenous entity of PDAC [18,19,20]. This notion is reinforced by the fact that histopathological samples are by default a significant underrepresentation of the tumor, since they are derived from a small sub-section of the tissue [21], and regions of differing molecular subtype are likely to coexist within the same tumor [22]. The exact nature of the so-called unclassifiable subtype might thus be presumed to represent a tumor simultaneously exposing a dual phenotype or a transitional phenotype on a continuum between full HNF1a and KRT81 positivity, mirrored in the intermediate survival outcome, both in our own and in previous studies [3]. A more fine-grained analysis would benefit from a global, i.e., whole tumor quantitative analysis of KRT81 and HNF1a expression, which is currently unfeasible due to the necessity of whole tumor work-up. Furthermore, such classification would result in small subgroup sizes, rendering the machine-learning analysis impossible in the current study.

The potential benefit of a radiomic workflow consequently lies in volumetric whole-tumor assessment, providing an opportunity to establish a clinically relevant phenotyping system and to better inform precision therapy regimens, and this concept of correlating quantitative morphometric evaluation with molecular phenotypes has been recently demonstrated for PDAC [23].

We previously presented a machine learning-algorithm capable of subtype characterization and—by extension—patient survival, based on pre-operative diffusion weighted MRI [8,9]. Extending this work, our current findings successfully transfer this methodology to routine CT acquisitions. The benefits of CT include broad availability, fewer motion artifacts and high standardization. We included CT images from several vendors, both to maximize sample size and to prompt vendor-invariance of the algorithm [17].

The process of Radiomics is dependent on source data standardization, pre-processing and can suffer from limited reproducibility, especially across modalities [24]. However, in our previous work and in other authors’ work, heterogeneity-related features have been shown to be both prognostic and among the most reproducible [25], and it is reassuring to observe features belonging to this group, such as Entropy and Variance, re-surfacing in the current analysis. We therefore hypothesize that heterogeneity is a distinguishing feature of QM tumors and propose further investigation and development of specific heterogeneity imaging biomarkers.

Our study suffers from several limitations: the dataset in our study is unlikely to allow broad generalization due to its limited size, class imbalance and retrospective mono-institutional nature, necessitating cross-validation, which may encourage overfitting and yield overly optimistic classification metrics. This lack of multi-institutional prospective validation and datasets of sufficient statistical power is a notable issue with many radiomic studies [26]. Furthermore, any definition of PDAC subtypes may suffer from label noise, and it cannot be conclusively resolved at this point, how robustly the applied histopathological methodology can represent the transcriptome-based molecular phenotype [22]. Nevertheless, the highly significant separation of survival in the unclassifiable tumor subgroup observed in our study seems to support the binary classification of PDAC subtypes as recently proposed [27].

## 5. Conclusions

In conclusion, our study represents an iterative evolution of previously developed methods of radiomic phenotyping of PDAC and should be expanded and validated in larger retrospective and prospective study settings.

## Figures and Tables

**Table 1:** Clinical parameters and cross-tabulation results for the QM, non-QM and unclassifiable cohorts. Abbreviations: pT: tumor T-stage, pN: nodal status, M: metastasis, G: histopathological grading, R: resection margins (All UICC 6th ed.), CA19-9 and CEA: Carbohydrate Antigen 19-9 and Carcinoembryonic Antigen, N.A.: Not available. Statistical tests used: 1: Chi-Squared-Test, 2: one-way ANOVA, 3: Log-Rank-Test. N.S.: Not significant at the two-sided level of *p* < 0.05.

	Variable	QM (*n* = 45)	Non-QM (*n* = 136)	Unclassifiable (*n* = 26)	*p*-Value
**Sex**	Male Female	25 (55%) 20 (45%)	75 (55%) 61 (45%)	14 (54%) 12 (46%)	0.99 ^1^
**Age** **(years)**	Mean Range	68 42–87	67 45–90	72 53–90	0.84 ^2^
**pT**	T1 T2 T3 T4	1 (2%) 8 (18%) 31 (69%) 5 (11%)	3 (2%) 19 (14%) 103 (76%) 11(8%)	1(4%) 4 (15%) 18 (69%) 3 (12%)	0.97 ^1^
**pN**	N0 N1	13 (29%) 32 (71%)	31 (23%) 105 (77%)	5 (19%) N1 (81%)	0.60 ^1^
**M**	M0 M1	39 (87%) 6 (13%)	125 (91%) 11 (8%)	25 (96%) 1 (4%)	0.36 ^1^
**G**	G1 G2 G3	1 (2%) 21 (47%) 23 (51%)	6 (4%) 60 (45%) 70 (51%)	4 (15%) 13 (50%) 9 (35%)	0.11 ^1^
**R**	R0 R1	20 (44%) 25 (56%)	68 (50%) 68 (50%)	15 (58%) 11 (42%)	0.56 ^1^
**CA19-9**	Normal Elevated N.A.	5 (10%) 20 (45%) 20 (45%)	22 (16%) 43 (32%) 71 (52%)	2 (8%) 5 (19%) 19 (73%)	0.11 ^1^
**CEA**	Normal Elevated N.A.	12 (27%) 8 (18%) 25 (55%)	38 (27%) 12 (9%) 86 (64%)	3 (11%) 1(4%) 22 (85)	0.08 ^1^
**First-Line** **Chemotherapy**	Gemcitabine FOLFIRINOX Did not receive	16 (36%) 3 (7%) 26 (57%)	68 (50%) 2 (2%) 66 (48%)	12 (46%) 2 (8%) 12 (46%)	0.16 ^1^
**Median Overall Survival (months)**		9.5	16.5	14.6	- QM vs. Non-QM: 0.03 ^3^ - Others N.S. ^3^
**Censored**	No Yes	31 (69%) 14 (31%)	97 (71%) 39 (29%)	20 (77%) 6 (23%)	0.77 ^1^
**Tumor Location**	Head/Body Tail	44 (98%) 1 (2%)	133 (98%) 3 (2%)	25 (96%) 1 (4%)	0.87 ^1^

## Supplementary Materials

The following are available online at https://www.mdpi.com/2077-0383/9/3/724/s1: Biostatistics analyses, details on the radiomic extraction process, the machine learning modelling and the radiomic parameters, alongside technical evaluation of the study and the STROBE checklist.
