# Supplementary material for: Image-Based Molecular Phenotyping of Pancreatic Ductal Adenocarcinoma
Source: J Clin Med. 2020 Mar 7;9(3):724. doi: 10.3390/jcm9030724 (PMC7141256; doi:10.3390/jcm9030724)
Supplement: Supplementary file 1 [file jcm-09-00724-s001.pdf]

Image-based molecular phenotyping of pancreatic ductal adenocarcinoma

Table of Contents

	Page
Supplementary Figure S1	2
Supplementary Figure S2	3
Radiomics Extraction Process and Machine Learning Modelling	4
Supplementary Table S1	5
Technical Evaluation of the Study according to RSNA criteria	6
STROBE Statement checklist	8
Patient inclusion flowchart	10
Supplemental References	11

Georgios A. Kaissis, Sebastian Ziegelmayer, Fabian K. Lohöfer, Felix N. Harder, Friederike Jungmann, Daniel Sasse, Alexander Muckenhuber, Hsi-Yu Yen, Katja Steiger, Jens Siveke, Helmut Friess, Roland Schmid, Wilko Weichert, Marcus R. Makowski and Rickmer F. Braren

Supplement

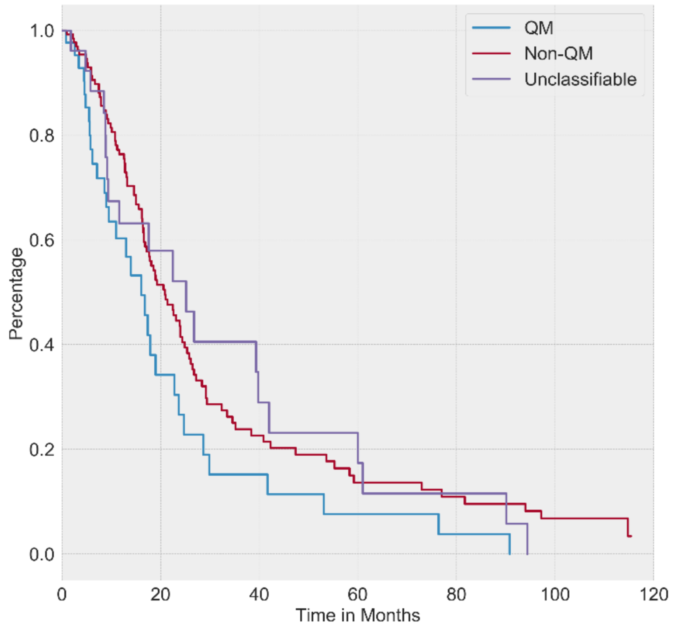

Supplementary Figure S1:

Patients with a histopathological label of *quasi-mesenchymal*, QM) (blue curve) experienced significantly diminished overall survival compared to patients with a histopathological label of non-QM (red curve), (16.1 vs. 20.9 months median OS, log-rank-test p=0.02, HR 1.59, 95% CI 1.08-2.53). Patients with an *unclassifiable* histopathological phenotype experienced a median overall survival time of 25.2 months, and the survival curves crossed both other groups' curves, leading to breach of the proportional hazards assumptions and statistical non-significance (p=0.18 for KRT81+ vs. *unclassifiable* and p=0.97 for HNF1a+ vs. *unclassifiable*).

Parameter	*exp(coef)*	*exp(coef) upper 95%*	*exp(coef) upper 95%*	*p*
pT	1.46	2.138	2.138	0.052
pN	1.438	2.084	2.084	0.055
G	1.31	1.763	1.763	0.074
CA199	1.221	1.541	1.541	0.092
cM	1.694	3.146	3.146	0.095
adjuvant	0.857	1.068	1.068	0.169
CEA	0.795	1.131	1.131	0.202
Age	1.009	1.026	1.026	0.258
Sex	1.08	1.574	1.574	0.687
R	1.029	1.521	1.521	0.884

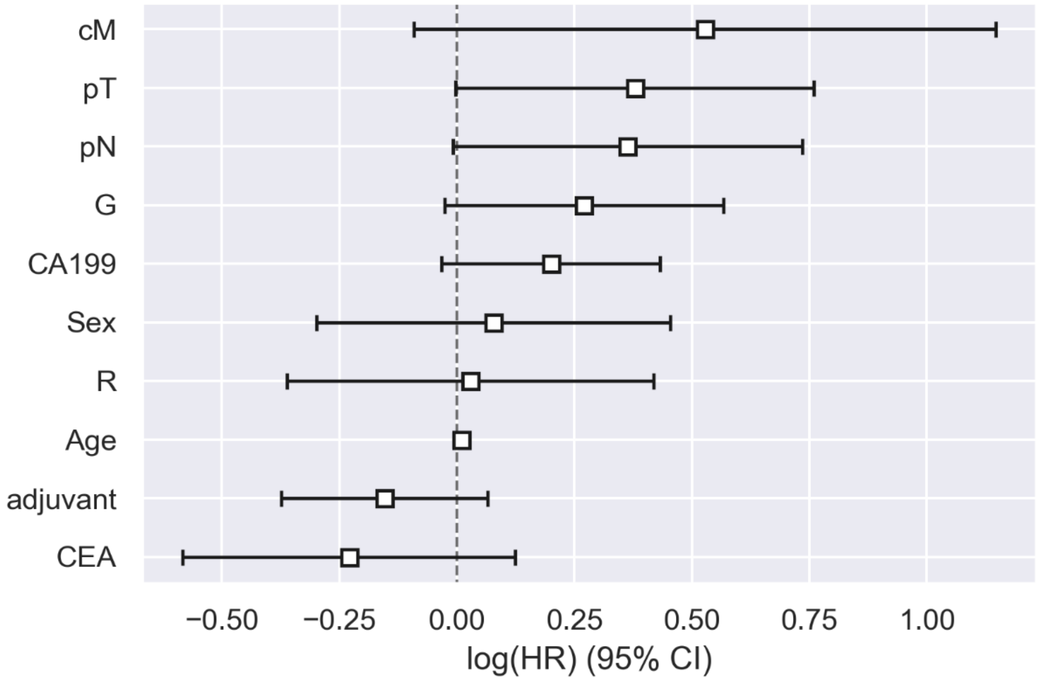

Supplementary Figure S2:

Cox proportional hazards multivariate survival analysis did not yield any significant survival covariate in the cohort A patients.

Radiomics Extraction Process and Machine Learning Modelling

PyRadiomics version 2.1.0 [1] was used for the analysis. Intensity discretization was performed to a fixed bin number of 25 bins. No normalization was performed. Images were spatially resampled to 3x3x3mm using the *BSpline* interpolator. All first order statistics, shape-based, Gray Level Run Length Matrix, Gray Level Size Zone Matrix, Neighbouring Gray Tone Difference Matrix and Gray Level Dependence Matrix features and all Gray Level Cooccurence Matrix features except *SumAverage* (due to redundancy), as well as Laplacian of Gaussian-filtered (with Sigma values 1.0, 2.0, 3.0), wavelet-decomposition-based (using the coiflet 1 function), square, exponential, gradient, square-root and logarithm filtered versions of these features. GLCM and GLRLM were extracted using the default settings (separately for each direction then averaged). Feature descriptions can be found in the PyRadiomics documentation. 1474 features were extracted in total.
The following radiomic features were excluded: Features yielding nil, constant or-missing values. Furthermore, tumors were segmented a second time after 2 weeks by the same observers to test for repeated segmentation stability. The intra-class-correlation coefficient (two-way mixed effects model/ consistency as described by McGraw and Wong [2]) was calculated and features yielding inter-segmentation values below 0.9 were excluded.
Machine learning modeling was performed using the Python programming language version 3.7.6. For training and testing, the estimator was fit and tested using stratified shuffle/split cross-validation with 5 splits of 70%/30% (train/test) of the dataset. The Random Forest algorithm implemented in *Scikit Learn 0.21.3* was used with the following setting: 10 random trees, *Gini impurity* feature importance assessment, all-available-core parallelization (with 18 available CPU cores). The other settings were left at default values.

Supplementary Table S1. Most important radiomic features selected by the Random Forest algorithm with respect to classification performance in descending order.

Radiomic Feature	***Average Feature Importance***	***STDEV***
wavelet-HLL_firstorder_Uniformity	0.056	0.028
wavelet-LHL_firstorder_TotalEnergy	0.048	0.072
wavelet-HHH_glcm_Id	0.046	0.056
wavelet-HHL_firstorder_10Percentile	0.042	0.035
wavelet-HHL_gldm_LowGrayLevelEmphasis	0.04	0.033
original_glcm_Idm	0.039	0.031
wavelet-HHH_glcm_Idm	0.039	0.051
wavelet-HLL_glcm_InverseVariance	0.035	0.029
wavelet-HLL_glrlm_LongRunLowGrayLevelEmphasis	0.034	0.06
wavelet-HLL_glszm_GrayLevelNonUniformityNormalized	0.028	0.049
wavelet-HLH_glcm_Correlation	0.025	0.031
wavelet-HHH_glcm_JointEnergy	0.025	0.031
wavelet-LLH_glcm_DifferenceVariance	0.025	0.045
wavelet-HLL_glszm_LargeAreaLowGrayLevelEmphasis	0.024	0.026
wavelet-HHL_glrlm_ShortRunLowGrayLevelEmphasis	0.018	0.028
wavelet-HLL_glcm_DifferenceEntropy	0.015	0.024
logarithm_gldm_SmallDependenceLowGrayLevelEmphasis	0.015	0.028
wavelet-LHL_firstorder_10Percentile	0.014	0.02
wavelet-HLL_firstorder_Energy	0.013	0.023
log-sigma-2-0-mm-3D_gldm_DependenceVariance	0.012	0.024

Technical Evaluation of the Study according to RSNA criteria

The Radiological Society of North America recently published evaluation criteria for artificial intelligence studies in radiology [3] and recommends adherence to these criteria for all such studies. The point-to-point assessment of our study is detailed below:

1. Carefully define all three image sets (training, validation, and test sets of images) of the AI experiment.

The training and validation sets used in the study were derived by cross-validation of the original set of 181 patients to ascertain that no validation data leaks into the training set in each fold, although the employed shuffle-split cross-validation method does not guarantee non-overlapping splits. The test set was completely independent and consisted of 26 patients. Patients were included based on availability of technically sufficient CT scans as detailed in the main manuscript. No outliers were removed.

2. Use an external test set for final statistical reporting.

and

3. Use multivendor images, preferably for each phase of the AI evaluation (training, validation, test sets).

The final assessment of the algorithm did not occur on an external test set. However, we pooled image datasets from several CT scanner vendors to provide robustness against this variable. The high degree of standardization in computed tomography likely also provides a level of multi-vendor generalizability. Despite this, the generalization power of the algorithm cannot be conclusively assessed at this stage.

4. Justify the size of the training, validation, and test sets.

The size of the training, validation and test sets were determined by the number of patients who had undergone histopathological evaluation to obtain a label for algorithm training as detailed in the main manuscript. Since this histopathological evaluation is both costly and time-consuming, the ultimate sample size could not be further increased.

5. Train the AI algorithm using a standard of reference that is widely accepted in our field.

The algorithm was trained on histopathological labels, which are a published standard of reference valid for assessment of molecular PDAC subtype (see reference in main manuscript).

6. Describe any preparation of images for the AI algorithm.

The images were prepared using a state-of-the art approach (independent segmentations with quality control, discarding unstable features, feature reduction, preprocessing and analysis using standardized and open-source software). No other manipulation of the source images occured.

7. Benchmark the AI performance to radiology experts

We did not perform formal benchmarking as part of the study but will perform a separate study on benchmarking human observers against the algorithm. However, expert observers noted no visual differences between the CT images of KRT81+ vs. HNF1a+ tumors.

8. Demonstrate how the AI algorithm makes decisions.

Interpretability of Random Forest models is provided by the inbuilt feature importance metric, which is reported in detail in the main manuscript.

9. The AI algorithm should be publicly available so that claims of performance can be verified

Source code for the analysis will be made available after publication on the author's GitHub page under a permissive open-source license and using the open-source Python programming language. A binary implementation of the algorithm will be included.

STROBE checklist and patient recruitment flowchart

	Item No	Reccomendation	Remark/ Location
**Title and abstract**	1	(*a*) Indicate the study's design with a commonly used term in the title or the abstract	Reported in abstract (*Methods)*
		(*b*) Provide in the abstract an informative and balanced summary of what was done and what was found	Reported in abstract (*Methods, Results)*
**Introduction**			
Background/rationale	2	Explain the scientific background and rationale for the investigation being reported	Abstract, Introduction
Objectives	3	State specific objectives, including any prespecified hypotheses	Introduction, Discussion
**Methods**			
Study design	4	Present key elements of study design early in the paper	Methods
Setting	5	Describe the setting, locations, and relevant dates, including periods of recruitment, exposure, follow-up, and data collection	Ibid.
Participants	6	(*a*) Give the eligibility criteria, and the sources and methods of selection of participants. Describe methods of follow-up	Ibid.
		(*b*) For matched studies, give matching criteria and number of exposed and unexposed	Not applicable
Variables	7	Clearly define all outcomes, exposures, predictors, potential confounders, and effect modifiers. Give diagnostic criteria, if applicable	Methods, Results
Data sources/ measurement	8*	For each variable of interest, give sources of data and details of methods of assessment (measurement). Describe comparability of assessment methods if there is more than one group	Methods
Bias	9	Describe any efforts to address potential sources of bias	Methods, Results
Study size	10	Explain how the study size was arrived at	Methods/ Supplement
Quantitative variables	11	Explain how quantitative variables were handled in the analyses. If applicable, describe which groupings were chosen and why	Methods, Table 1
Statistical methods	12	(*a*) Describe all statistical methods, including those used to control for confounding	Methods
		(*b*) Describe any methods used to examine subgroups and interactions	Ibid.
		(*c*) Explain how missing data were addressed	Methods, Table 1
		(*d*) If applicable, explain how loss to follow-up was addressed	Methods, Table 1
		(*e*) Describe any sensitivity analyses	Not applicable
**Results**			
Participants	13*	(a) Report numbers of individuals at each stage of study—eg numbers potentially eligible, examined for eligibility, confirmed eligible, included in the study, completing follow-up, and analysed	Methods, Supplementary Material

		(b) Give reasons for non-participation at each stage	Ibid.
		(c) Consider use of a flow diagram	See below
Descriptive data	14*	(a) Give characteristics of study participants (eg demographic, clinical, social) and information on exposures and potential confounders	Methods, Table 1, Results, Supplement
		(b) Indicate number of participants with missing data for each variable of interest	Methods, Table 1
		(c) Summarise follow-up time (eg, average and total amount)	Methods
Outcome data	15*	Report numbers of outcome events or summary measures over time	Results, Supplement
Main results	16	(*a*) Give unadjusted estimates and, if applicable, confounder-adjusted estimates and their precision (eg, 95% confidence interval). Make clear which confounders were adjusted for and why they were included	Results, Supplement
		(*b*) Report category boundaries when continuous variables were categorized	Not applicable
		(*c*) If relevant, consider translating estimates of relative risk into absolute risk for a meaningful time period	Not applicable
Other analyses	17	Report other analyses done—eg analyses of subgroups and interactions, and sensitivity analyses	Results, Supplement
**Discussion**			
Key results	18	Summarise key results with reference to study objectives	Discussion
Limitations	19	Discuss limitations of the study, taking into account sources of potential bias or imprecision. Discuss both direction and magnitude of any potential bias	Discussion
Interpretation	20	Give a cautious overall interpretation of results considering objectives, limitations, multiplicity of analyses, results from similar studies, and other relevant evidence	Discussion
Generalisability	21	Discuss the generalisability (external validity) of the study results	Discussion Supplementary Material
**Other information**			
Funding	22	Give the source of funding and the role of the funders for the present study and, if applicable, for the original study on which the present article is based	Preamble

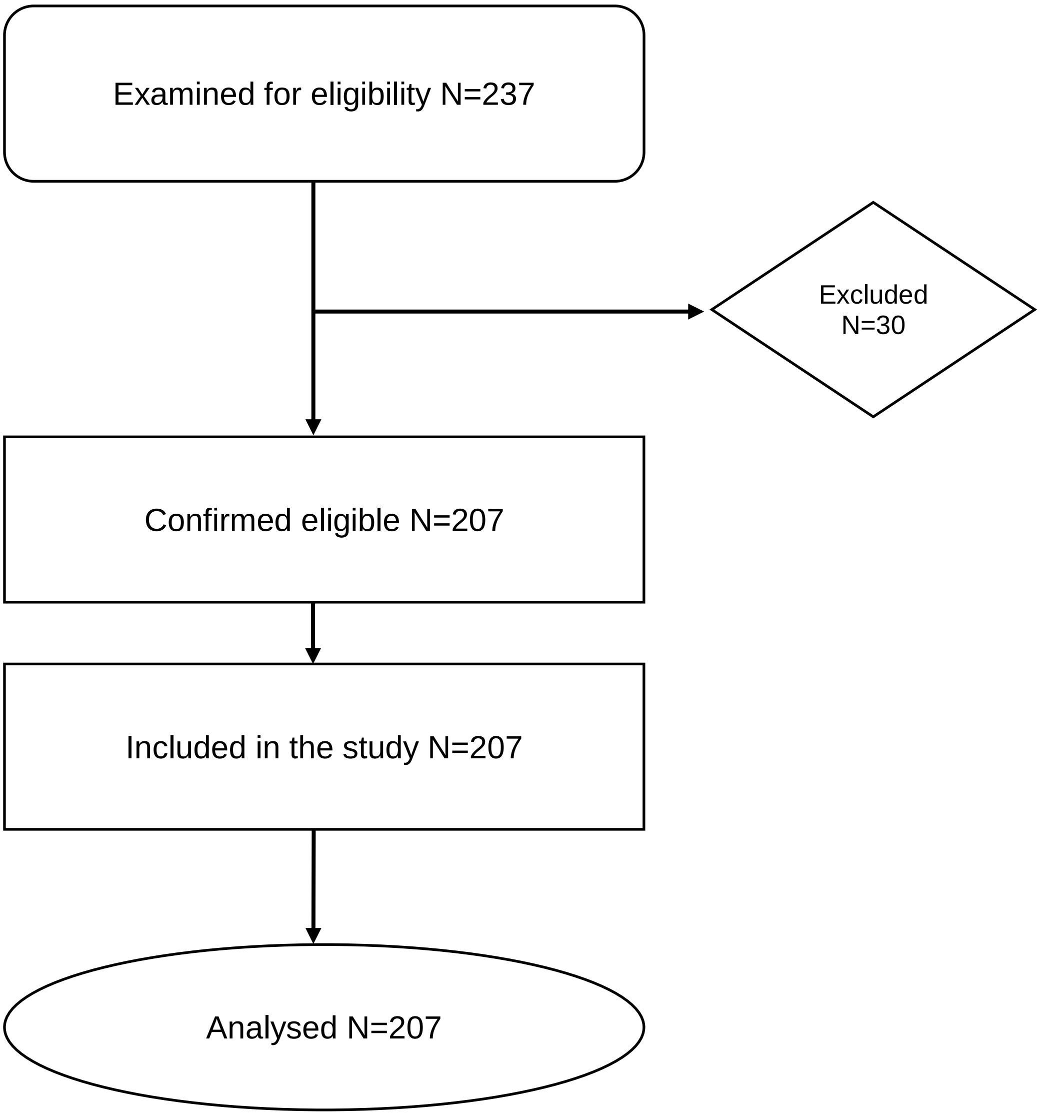

Reasons for exclusion:
Received prior treatment (N= 9)
Insufficient technical quality (N= 6)
Pre-existent active malignant disease (N=3)
Loss to follow-up earlier than 2-weeks postoperatively (N= 12)

Supplemental References

[1] Griethuysen, J. J. M., Fedorov, A., Parmar, C., Hosny, A., Aucoin, N., Narayan, V., Beets-Tan, R. G. H., Fillon-Robin, J. C., Pieper, S., Aerts, H. J. W. L. (2017). Computational Radiomics System to Decode the Radiographic Phenotype. Cancer Research, 77(21), e104–e107. `https://doi.org/10.1158/0008-5472.CAN-17-0339 https://doi.org/10.1158/0008-5472.CAN-17-0339

[2] McGraw, K. O., & Wong, S. P. (1996). Forming inferences about some intraclass correlation coefficients. *Psychological Methods, 1*(1), 30-46.

[3] Assessing Radiology Research on Artificial Intelligence: A Brief Guide for Authors, Reviewers, and Readers—From the Radiology Editorial Board
David A. Bluemke, Linda Moy, Miriam A. Bredella, Birgit B. Ertl-Wagner, Kathryn J. Fowler, Vicky J. Goh, Elkan F. Halpern, Christopher P. Hess, Mark L. Schiebler, and Clifford R. Weiss, https://doi.org/10.1148/radiol.2019192515
